# Multiple approaches for massively parallel sequencing of SARS-CoV-2 genomes directly from clinical samples

**DOI:** 10.1186/s13073-020-00751-4

**Published:** 2020-06-30

**Authors:** Minfeng Xiao, Xiaoqing Liu, Jingkai Ji, Min Li, Jiandong Li, Lin Yang, Wanying Sun, Peidi Ren, Guifang Yang, Jincun Zhao, Tianzhu Liang, Huahui Ren, Tian Chen, Huanzi Zhong, Wenchen Song, Yanqun Wang, Ziqing Deng, Yanping Zhao, Zhihua Ou, Daxi Wang, Jielun Cai, Xinyi Cheng, Taiqing Feng, Honglong Wu, Yanping Gong, Huanming Yang, Jian Wang, Xun Xu, Shida Zhu, Fang Chen, Yanyan Zhang, Weijun Chen, Yimin Li, Junhua Li

**Affiliations:** 1 BGI-Shenzhen, Shenzhen, 518083 China; 2 Shenzhen Key Laboratory of Unknown Pathogen Identification, BGI-Shenzhen, Shenzhen, 518083 China; 3 State Key Laboratory of Respiratory Disease, National Clinical Research Center for Respiratory Disease, Guangzhou Institute of Respiratory Health, the First Affiliated Hospital of Guangzhou Medical University, Guangzhou, China; 4 School of Future Technology, University of Chinese Academy of Sciences, Beijing, 101408 China; 5 BGI Education Center, University of Chinese Academy of Sciences, Shenzhen, 518083 China; 6 MGI, BGI-Shenzhen, Shenzhen, 518083 China; 7 Institute of Infectious Disease, Guangzhou Eighth People’s Hospital of Guangzhou Medical University, Guangzhou, China; 8 School of Biology and Biological Engineering, South China University of Technology, Guangzhou, China; 9 BGI PathoGenesis Pharmaceutical Technology, Shenzhen, China; 10 James D. Watson Institute of Genome Science, Hangzhou, 310008 China; 11 Guangdong Provincial Key Laboratory of Genome Read and Write, BGI-Shenzhen, Shenzhen, 518120 China; 12 Shenzhen Engineering Laboratory for Innovative Molecular Diagnostics, BGI-Shenzhen, Shenzhen, 518120 China

**Keywords:** Emerging infectious diseases, COVID-19, Metatranscriptomic sequencing, Hybrid capture, Multiplex PCR, iSNV, Quasispecies, Genomic surveillance, Virus evolution

## Abstract

**Background:**

COVID-19 (coronavirus disease 2019) has caused a major epidemic worldwide; however, much is yet to be known about the epidemiology and evolution of the virus partly due to the scarcity of full-length SARS-CoV-2 (severe acute respiratory syndrome coronavirus 2) genomes reported. One reason is that the challenges underneath sequencing SARS-CoV-2 directly from clinical samples have not been completely tackled, i.e., sequencing samples with low viral load often results in insufficient viral reads for analyses.

**Methods:**

We applied a novel multiplex PCR amplicon (amplicon)-based and hybrid capture (capture)-based sequencing, as well as ultra-high-throughput metatranscriptomic (meta) sequencing in retrieving complete genomes, inter-individual and intra-individual variations of SARS-CoV-2 from serials dilutions of a cultured isolate, and eight clinical samples covering a range of sample types and viral loads. We also examined and compared the sensitivity, accuracy, and other characteristics of these approaches in a comprehensive manner.

**Results:**

We demonstrated that both amplicon and capture methods efficiently enriched SARS-CoV-2 content from clinical samples, while the enrichment efficiency of amplicon outran that of capture in more challenging samples. We found that capture was not as accurate as meta and amplicon in identifying between-sample variations, whereas amplicon method was not as accurate as the other two in investigating within-sample variations, suggesting amplicon sequencing was not suitable for studying virus-host interactions and viral transmission that heavily rely on intra-host dynamics. We illustrated that meta uncovered rich genetic information in the clinical samples besides SARS-CoV-2, providing references for clinical diagnostics and therapeutics. Taken all factors above and cost-effectiveness into consideration, we proposed guidance for how to choose sequencing strategy for SARS-CoV-2 under different situations.

**Conclusions:**

This is, to the best of our knowledge, the first work systematically investigating inter- and intra-individual variations of SARS-CoV-2 using amplicon- and capture-based whole-genome sequencing, as well as the first comparative study among multiple approaches. Our work offers practical solutions for genome sequencing and analyses of SARS-CoV-2 and other emerging viruses.

## Background

As of 14 March 2020, severe acute respiratory syndrome coronavirus 2 (SARS-CoV-2) has surpassed severe acute respiratory syndrome coronavirus (SARS-CoV) and Middle East respiratory syndrome coronavirus (MERS-CoV) in every aspect, infecting over 140,000 people in more than 110 countries, with a mortality of over 5000 [1]. So far, coronaviruses have caused three major epidemics in the past two decades, posing a great challenge to global health and economy. Massively parallel sequencing (MPS) of viral genomes has demonstrated enormous capacity as a powerful tool to study emerging infectious diseases, such as SARS, MERS, Zika, and Ebola, in tracing the outbreak origin and drivers, tracking transmission chains, mapping the spread, and monitoring the evolution of the etiological agents [2–7]. Though by 14 March 2020, fewer than 500 SARS-CoV-2 genomes were published on public databases including China National GeneBank DataBase (CNGBdb), NCBI GenBank, and the Global Initiative on Sharing All Influenza Data (GISAID), and much remains unknown about the epidemiology and evolution of the virus. One possible explanation of the paucity of published SARS-CoV-2 genomes was the challenges posed by sequencing clinical samples with low virus abundance.

The first teams obtained the SARS-CoV-2 genome sequences through metatranscriptomic MPS, supplemented by PCR and Sanger sequencing of a combination of bronchoalveolar-lavage fluid (BALF) and culture [8–10] or from BALF directly [11]. Experience from studying SARS-CoV showed that BALF from the lower respiratory tract was an ideal sample type with higher viral load [12]. However, BALF was not routinely collected from every patient, and human airway epithelial (HAE) cell culture is very labor-intensive and time-consuming, taking 4 to 6 weeks [9, 13]. Chan et al. managed to get the whole-genome sequences through metatranscriptomic sequencing with Oxford Nanopore platform supplemented by Sanger sequencing from both nasopharyngeal and sputum specimens after single-primer amplification [14]. Holshue et al. published the whole-genome sequence using oropharyngeal and nasopharyngeal specimens through Sanger and metatranscriptomic sequencing with both Illumina and MinIon [15]. To date, multiplex PCR-based or hybrid capture-based whole-genome sequencing of SARS-CoV-2, as well as comparative studies between different approaches, have not been reported on peer-reviewed journals.

Besides inter-individual variations, dissecting intra-individual dynamics of viruses also largely promotes our understanding of virus-host interactions, viral evolution, and transmission as demonstrated for Ebola, Zika, Influenza, etc. [5, 16–18]. The analyses of intra-individual single nucleotide variations (iSNVs) and its allele frequency have also contributed to anti-viral therapy and drug resistance, e.g., to reveal highly conserved genes during the outbreak that potentially serve as ideal therapeutic targets [17, 19]. However, it is challenging to accurately detect iSNVs from clinical samples, especially when the samples are subjected to extra steps of enrichment and amplification.

Therefore, we aim to comprehensively compare the sensitivity, inter-individual (variant) and intra-individual (iSNV) accuracy, and other general features of different approaches by systematically utilizing ultra-high-throughput metatranscriptomic, hybrid capture-based, and amplicon-based sequencing approaches to obtain genomic information of SARS-CoV-2 from serial dilutions of a cultured isolate and directly from clinical samples. We present a reasonable sequencing strategy that fits into different scenarios and estimate the minimal amount of sequencing data for downstream SARS-CoV-2 genome analyses. Our study offers practical solutions to facilitate the studies of SARS-CoV-2 and other emerging viruses in the future and would promote extensive genomic sequencing and analyses of SARS-CoV-2 and other emerging viruses, which would in turn contribute to real-time virus surveillance and managing viral outbreaks. Benefiting from our experimental workflows and bioinformatic pipelines, BGI Group has launched a Global Initiative on Open-source Genomics for SARS-CoV-2 (GIOG-S, https://giogs.genomics.cn/) and makes its platforms for multiplex PCR sequencing and ultra-deep metatranscriptomic sequencing available to global research teams within GIOG-S.

## Methods

### Sampling, RNA extraction, reverse transcription, and qRT-PCR

Clinical specimens (including throat swab, nasal swab, anal swab, and sputum) were obtained from confirmed COVID-19 cases at the First Affiliated Hospital of Guangzhou Medical University. Total RNA of the cultured isolate of SARS-CoV-2 was obtained from the Academy of Military Medical Science (AMMS) and subjected to 10-fold serial dilutions. Virus isolation and RNA extraction were done in a biosafety level (BSL) 2+ laboratory with BSL-3 protection. Total RNA was extracted directly from the clinical specimens without inactivating the virus with QiAamp RNeasy Mini Kit (Qiagen, Heiden, Germany) following the manufacturer’s instructions without modification. Real-time reverse transcription PCR (qRT-PCR) targeting RdRp gene and N gene of SARS-CoV-2 was used to detect and quantify the viral RNA within clinical samples and serial dilutions of the cultured isolate using the SARS-CoV-2 Nucleic Acid Detection Kit following the manufacture’s protocol (Geneodx, Shanghai, China, and BGI-Shenzhen, Shenzhen, China).

### Metatranscriptomic library preparation and sequencing

Host DNA was removed from RNA samples using DNase I, and the concentration of RNA samples was measured by Qubit RNA HS Assay Kit (Thermo Fisher Scientific, Waltham, MA, USA). DNA-depleted and purified RNA was used to construct the double-stranded (ds) circular DNA library with MGIEasy RNA Library preparation reagent set (MGI, Shenzhen, China), as follows: (1) RNA was fragmented by incubating with fragmentation buffer at 87 °C for 6 min; (2) ds cDNA was synthesized using random hexamers with fragmented RNA; (3) ds cDNA was subjected to end repair, adaptor ligation, and 18-cycle PCR amplification; and (4) PCR products were unique dual indexed (UDI), before going through circularization and rolling circle replication (RCR) to generate DNA nanoball (DNB)-based libraries. Negative controls prepared from nuclease-free water and total RNA isolated from human Michigan Cancer Foundation-7 (MCF-7) breast cancer cells were included. DNB preps of clinical samples were sequenced on the ultra-high-throughput DNBSEQ-T7 platform (MGI, Shenzhen, China) with paired-end 100 nt strategy, generating 321 Gb sequencing data for each sample on average.

Outside of mainland China, BGI (https://www.bgi.com/global/) has regional headquarters in Europe (Copenhagen, Denmark), Asia Pacific (Hong Kong, China), and Americas (San Jose, CA, USA) and has been actively serving customers in more than 66 countries. MGI (https://en.mgitech.cn/) operates in 39 countries and regions with branches including Hong Kong in China, Kobe in Japan, Dubai in UAE, Riga in Latvia, and San Jose in the USA. The global training and service network is located in major countries and regions on 6 continents, with 40 training/after-sales service centers. To acquire reagents, instruments, and technical support, researchers may find regional contact from the official website or directly send enquiries to the specified corresponding author Y.Z. (zhangyanyan@genomics.cn).

### Hybrid capture-based enrichment and sequencing

A hybrid capture technique was used to enrich SARS-CoV-2-specific content from the metatranscriptomic double-stranded DNA libraries with the 2019-nCoVirus DNA/RNA Capture Panel (BOKE, Beijing, China). Negative controls prepared from nuclease-free water and total RNA isolated from human MCF-7 breast cancer cells were included. The manufacturer’s instructions were slightly modified to accommodate the MGISEQ-2000 platform, i.e., blocker oligos and PCR primer oligos were replaced by MGIEasy exon capture assistive kit (MGI, Shenzhen, China). DNB-based libraries were constructed and sequenced on the MGISEQ-2000 platform with paired-end 100 nt strategy using the same protocol described above, generating 37 Gb sequencing data for each sample on average.

### Amplicon-based enrichment and sequencing

Total RNA was reverse transcribed to synthesize the first-strand cDNA with random hexamers and Superscript II reverse transcriptase kit (Invitrogen, Carlsbad, USA). Sequencing was attempted on all samples regardless of Ct value including negative controls prepared from nuclease-free water and NA12878 human gDNA. A two-step SARS-CoV-2 genome amplification was performed with an equimolar mixture of primers using ATOPlex SARS-CoV-2 Full Length Genome Panel following the manufacture’s protocol (MGI, Shenzhen, China), generating 137× ~ 400 bp amplicons or 299× ~ 200 bp amplicons, and the genome positions of the amplicons are shown in Additional file 1 Table S1. Twenty microliters of first-strand cDNA was mixed with the components of the first PCR reaction following the manufacturer’s instructions. Two nanograms of human gDNA was added to each PCR reaction of the cultured isolate. A set of controls was adopted to help quantifying viral load and identify potential contamination. During library construction for amplicon sequencing, each sample was mixed with a fixed copy number of lambda genomic DNA (external control), and the external control and the SARS-CoV-2 genomes were amplified at the same time. The PCR was performed as follows: 5 min at 37 °C; 10 min at 95 °C; 15 cycles of 10 s at 95 °C, 1 min at 64 °C, 1 min at 60 °C, to 10 s at 72 °C; and 2 min at 72 °C. The products were purified with MGI EasyDNA Clean beads (MGI, BGI-Shenzhen, China) at a 5:4 ratio and cleaned with 80% concentration ethanol according to the manufacturer’s instructions. The 2nd PCR was performed under the same regimen as the 1st PCR except for 25 cycles, and the bead-purified products from the first PCR reaction were unique dual indexed. After the 2nd PCR, products were purified following the same procedures as the 1st PCR and quantified using the Qubit dsDNA High Sensitivity assay on Qubit 3.0 (Life Technologies). PCR products of samples yielding sufficient material (> 5 ng/μl) were pooled at equimolar to a total DNA amount of 300 ng before converting to single-stranded circular DNA. DNB-based libraries were generated from 20 μl of single-stranded circular DNA pools and sequenced on the MGISEQ-2000 platform with single-end 400 nt strategy, generating 1.8 Gb sequencing data for each sample on average.

### Identification of Coronaviridae-like reads in massively parallel sequencing data

For metatranscriptomic and hybrid capture sequencing data, total reads were first processed using Kraken v0.10.5 [20] (default parameters) with a self-built database of Coronaviridae genomes (including SARS, MERS, and SARS-CoV-2 genome sequences downloaded from GISAID, NCBI, and CNGB) to identify Coronaviridae-like reads in a loose manner. fastp v0.19.5 [21] (parameters: -q 20 -u 20 -n 1 -l 50) and SOAPnuke v1.5.6 [22] (parameters: -l 20 -q 0.2 -E 50 -n 0.02 -5 0 -Q 2 -G -d) were used to remove low-quality reads, duplications, and adaptor contaminations. Low-complexity reads were then removed using PRINSEQ v0.20.4 [23] (parameters: -lc_method dust -lc_threshold 7). Samples that exhibited higher coverage and at least 10-fold higher average sequencing depth than negative controls were accepted for downstream analyses of inter- and intra-individual variations.

For amplicon sequencing data, SE400 reads were first processed with fastp v0.19.5 [21] (parameters: -q 20 -u 20 -n 1 -l 50) to remove low-quality reads and adaptor sequences. Primer sequences and the 21 nt upstream and downstream of primers within the reads were then trimmed with BAMClipper v1.1.1 [24] (parameters: -n 4 -u 21 -d 21). Reads with low-quality bases, adaptors, primers, and adjacent sequences completely removed as described above were considered as clean reads for downstream analyses. The viral load of SARS-CoV-2 was quantified based on the data from both external control and the target virus. We define a *C* = target viral load/(target viral load + external control load), and a *C* > 0.1% of negative groups (prepared from human nucleic acids and nuclease-free water) indicates unacceptable contamination. Further, we consider a sample acceptable only when the *C* value of the sample is an order of magnitude higher than that of negative groups, for instance, if *C* < 0.01% for all negative controls and *C* > 0.1% for an experimental group.

### Genome assembly of SARS-CoV-2

For metatranscriptomic and hybrid capture sequencing, the Coronaviridae-like reads of samples with < 100× average sequencing depth were directly de novo assembled with SPAdes (v3.14.0, [25]) using the default settings. The Coronaviridae-like reads of samples with > 100× average sequencing depth across SARS-CoV-2 genome were subsampled to achieve 100× sequencing depth before being assembled.

For amplicon sequencing, SARS-CoV-2 consensus sequences were generated using Pilon v1.23 [26] (parameters: --changes –vcf --changes --vcf --mindepth 1 --fix all, amb). Nucleotide positions with sequencing depth < 100× or < 5-fold higher than that of negative controls were masked as ambiguous base N.

### Visualization of coverage depth across the viral genomes

The Coronaviridae-like reads from metatranscriptomic and hybrid capture sequencing data were aligned against the SARS-CoV-2 reference genome (GISAID accession: EPI_ISL_402119) [27] with BWA aln (v0.7.16) [28]. Duplications were identified by Picard Markduplicates (v2.10.10) (http://broadinstitute.github.io/picard) with default settings. For each sample, we calculated the depth of coverage at each nucleotide position of the SARS-CoV-2 reference genome with SAMtools (v1.9) [29] and scaled the values to the mean depth. For each nucleotide position, we calculated the median depth and 20th and 80th percentiles across all samples. Coverage depth across the SARS-CoV-2 reference genome was plotted within a 200-nt sliding window using the ggplot2 [30] package in R (v3.6.1) [31].

Amplicon sequencing data was processed as described above, except that duplications were not removed. A heatmap was generated to visualize the viral genome coverage for all samples sequenced by the amplicon method with the pheatmap v1.0.12 [32] package in R (v3.6.1) [31]. The depth at each nucleotide position was binarized and was shown in blue if depth of coverage was 100× and above.

### Relationship between viral load and minimum sequencing output across methods

SARS-CoV-2 reads of metatranscriptomic and hybrid capture sequencing data were identified by aligning the Coronaviridae-like reads against the SARS-CoV-2 reference genome (GISAID accession: EPI_ISL_402119) [27] with BWA in a strict manner of coverage ≥ 95% and identity ≥ 90%. For comparisons of the coverage depth of the viral genome across samples and methods, we normalized the viral reads to total sequencing reads with SARS-CoV-2 reads per million (SARS-CoV-2-RPM). SARS-CoV-2-RPM for amplicon sequencing data was calculated by the same pipeline applied for metatranscriptomic and hybrid capture sequencing data.

To estimate the minimum data required for genome assembly and genome variation analysis, we applied gradient-based sampling to the SARS-CoV-2 genome alignments (referred to BAM files) to each dataset using SAMtools (v1.9) [29]. The effective genome coverage was set as 95% for all three MPS methods. Considering the distinct technologies used in different methods, we set method-dependent thresholds of effective depth as follows: (1) ≥ 10× for metatranscriptomic sequencing, (2) ≥ 20× for hybrid capture sequencing, and (3) ≥ 100× for amplicon sequencing. We next calculated the coverage and depth within each subsampled BAM file per sample to determine the minimal BAM file that could meet the above thresholds of both coverage and sequencing depth. The method-dependent minimum sequencing output of each sample was estimated accordingly. We assessed the correlations between the SARS-CoV-2 genome copies per milliliter in diluted samples of cultured isolates and the minimum sequencing output for amplicon- and capture-based methods using Pearson’s correlation coefficient (*R*) with the function *scatter* from the R package (v3.6.1) *ggpubr* (v0.2.5) [33].

### Analysis of inter- and intra-individual variants

For metatranscriptomic and hybrid capture sequencing data, variant calling was performed based on the BAM files of identified SARS-CoV-2 reads after removing duplications using Picard Markduplicates (http://broadinstitute.github.io/picard). Amplicon sequencing data were processed as described above, except that duplications were not removed. Variants were first called with freebayes (v1.3.1) [34] (parameters: -p 1 -q 20 -m 60 --min-coverage 10 -V), and the low-confidence variants were removed with snippy-vcf_filter (v3.2) [35] (parameters: --minqual 100 --mincov 10 --minfrac 0.8). The remaining variants in VCF files generated by freebayes were annotated in SARS-CoV-2 genome assemblies and consensus sequences with SNVeff (v4.3) [36] using default parameters. Next, pysamstats v1.1.2 (https://github.com/alimanfoo/pysamstats) (parameters: -type variation_strand --min-baseq 20 -D 1000000) was used to count the number of matches, mismatches, deletions, and insertions at each base to determine the allele frequencies. Variant calls with allele frequencies ≥ 80% were identified as SNVs.

Nucleotide positions with ≥ 100× sequencing depth from amplicon sequencing, ≥ 10× from metatranscriptomic sequencing, and ≥ 20× from capture sequencing were kept for iSNV calling. The candidate iSNVs were further filtered as follows: (1) frequency filtering, only minor alleles (frequency ≥ 5% and < 50%) and major alleles (frequency ≥ 50% and ≤ 95%) were remained; (2) depth filtering, iSNVs with fewer than five forward or reverse reads were removed; and (3) strand bias filtering (not applicable to single-end reads of amplicon sequencing), iSNVs were removed if there were more than a 10-fold strand bias or a 5-fold difference between the strand bias of the variant call and that of the reference call.

### Taxonomy of clinical samples by unbiased metatranscriptomic sequencing

For metatranscriptomic sequencing of clinical samples, raw sequencing data of a single sequence lane (approximately 60–75 Gb per sample) was used to simultaneously assess the RNA expression patterns of human, bacteria, and viruses in clinical samples from COVID-19 patients. We first used the software fastp (v0.19.5) [21] to filter low-quality reads and remove adapter with parameters: -5 -3 -q 20 -c -l 30. After QC, we mapped high-quality reads to hg19 and removed human ribosomal RNA (rRNA) reads by using SOAP2 v2.21 [37] (parameters: -m 0 -x 1000 -s 28 -l 32 -v 5 -r 1), and the remaining RNA reads were then aligned to hg19 by HISAT2 [38] with default settings to identify non-rRNA human transcripts as previously described. Next, we applied Kraken 2 [39] (version 2.0.8-beta, parameters: --threads 24 --confidence 0) to assign microbial taxonomic ranks to non-human RNA reads against the large reference database MiniKraken2 (April 2019, 8 GB) built from the Refseq bacteria, archaea, and viral libraries and the h38 human genome. Bracken [40] (Bayesian Reestimation of Abundance with Kraken) was further applied to estimate microbial relative abundances based on taxonomic ranks of reads assigned by Kraken2.

## Results

### Design of the comparative study

We sampled eight specimens from COVID-19 patients in February 2020, including throat swab, nasal swab, anal swab, and sputum specimens, and the corresponding cycle threshold (Ct) value of SARS-CoV-2 qRT-PCR ranges from 18 to 32 (Table 1). We initially tried to boost the coverage and depth of the viral genome by ultra-deep metatranscriptomic sequencing with an average sequencing output of 1,607,264,105 paired-end reads (Table 1). Although we managed to obtain complete viral genome assemblies for each specimen, the sequencing depth varied across specimens. Only 0.002–0.003% of the total reads were assigned to the SARS-CoV-2 in three samples (GZMU0014, GZMU0030, and GZMU0031) with Ct between 29 and 32, resulting in inferior sequencing depth (less than 100×) (Table 1). Isolating viruses and enriching them in cell culture might improve the situation, but this requires high-standard laboratory settings and expertise apart from being time-consuming. Also, unexpected mutations that are not concordant with original clinical samples may be introduced during the culturing process.

**Table 1:** Metatranscriptomic sequencing data summary of eight SARS-CoV-2-positive clinical samples collected from Guangzhou in February 2020

Sample ID	Sample type	Ct	No. of sequencing read pairs	No. of SARS-CoV-2 read pairs	Percentage of SARS-CoV-2 read pairs	Coverage (%)	Depth (×)
**GZMU0047**	Nasal swab	18	1,547,648,648	85,316,930	5.513	100	113,021
**GZMU0016**	Sputum	21	1,578,573,142	7,489,563	0.474	99.96	12,734
**GZMU0048**	Throat swab	24	1,647,198,588	3,365,330	0.204	99.91	6508
**GZMU0044**	Nasal swab	26	1,609,367,415	7,275,402	0.452	99.92	12,758
**GZMU0030**	Throat swab	29	1,725,727,056	31,148	0.002	99.87	69
**GZMU0014**	Sputum	30	1,596,713,550	46,199	0.003	99.9	95
**GZMU0042**	Sputum	32	1,481,162,934	567,266	0.038	99.94	1133
**GZMU0031**	Anal swab	32	1,671,721,507	25,392	0.002	99.89	14

**Fig. 1:**
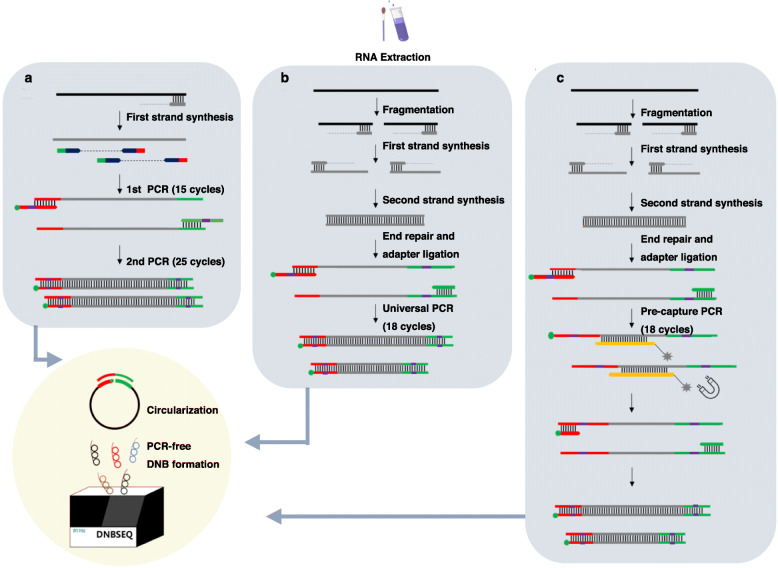
The general workflow of multiple sequencing approaches adopted in this study. We employed unique dual indexing (UDI) strategy and DNB-based (DNA nanoball) PCR-free MPS platform to minimize index hopping and relevant sequencing errors [41–43]. **a** Amplicon-based enrichment: the UDI was integrated in the 2nd PCR. Navy, multiplex PCR primers. **b** Metatranscriptomic library preparations: the UDI was integrated in the adaptor ligation and universal PCR steps. **c** Library preparations and hybrid capture-based enrichment: the UDI was integrated in the adaptor ligation and pre-capture PCR steps. Ocher, ssDNA probes. Red and green lines represent adaptor sequences; green dots represent phosphate groups

**Fig. 2:**
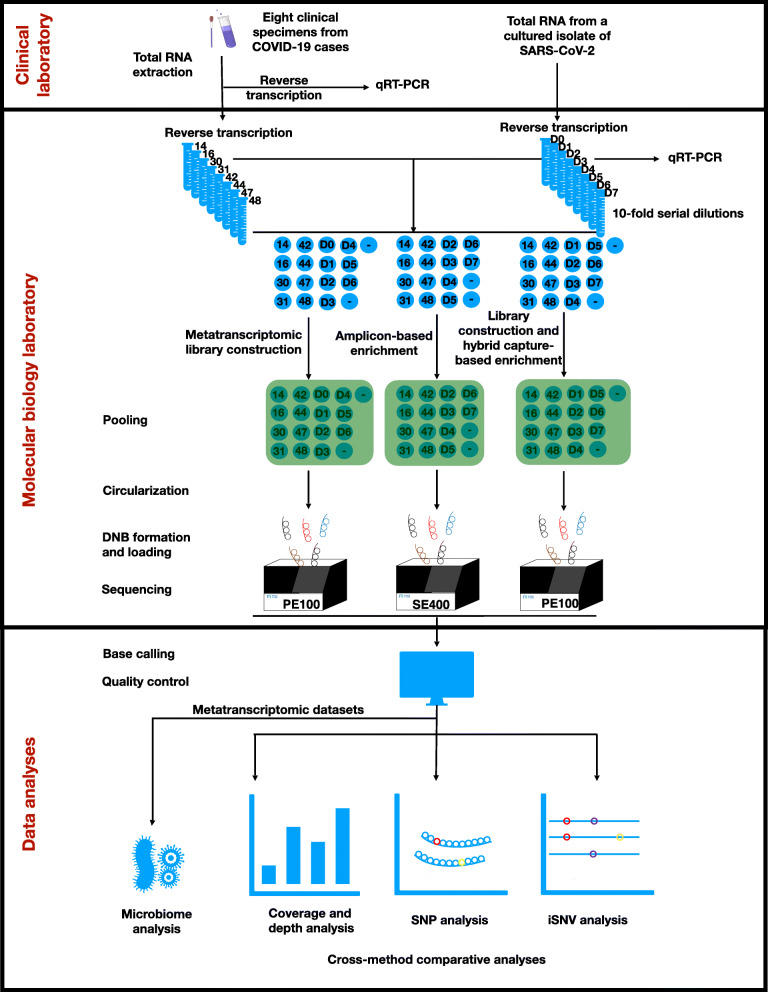
Overview of the study design. Eight clinical samples and serial dilutions of a cultured isolate were subjected to direct metatranscriptomic library construction, amplicon-based enrichment, and hybrid capture-based enrichment, respectively. Libraries generated from each method were pooled, respectively. DNB, DNA nanoball. 14, GZMU0014; 16, GZMU0016; 30, GZMU0030; 31, GZMU0031; 42, GZMU0042; 44, GZMU0044; 47, GZMU0047; 48, GZMU0048. D0, undiluted sample of the cultured isolate; D1–D7, seven serial diluted samples of the cultured isolate, ranging from 1E+07 to 1E+01 genome copies per milliliter, in 10-fold dilution. “-”, negative controls prepared from nuclease-free water and human nucleic acids. PE100, paired-end 100-nt reads; SE400, single-end 100-nt reads

To enrich adequate viral content for whole-genome sequencing in a convenient manner, we pursued two other methods: multiplex PCR amplification (amplicon) and hybrid capture (capture) (Fig. 1). We designed a systematic study to comprehensively validate the uniformity, sensitivity, and inter-individual (variant) and intra-individual (iSNV) accuracy of multiple approaches by sequencing serial dilutions of a cultured isolate (unpublished), as well as the eight clinical samples (Fig. 2). We performed qRT-PCR of 10-fold serial dilutions (D1–D7) of the cultured isolate, and the Ct was 17.3, 20.8, and 24.5 for 28.7, 31.8, 35, and 39.9, respectively, indicating the undiluted RNA (D0) of the cultured isolate contained ~ 1E+08 genome copies per milliliter. For amplicon sequencing, we utilized two kits comprising two sets of primers generating PCR products of 300–400 bp and 100–200 bp, respectively. The ~ 400 bp amplicon-based sequencing was implemented in all samples and analyzed throughout the study, while the ~ 200 bp amplicon-based sequencing was only applied in the cultured isolate for coverage analysis.

### Comparison of uniformity and sensitivity

**Fig. 3:**
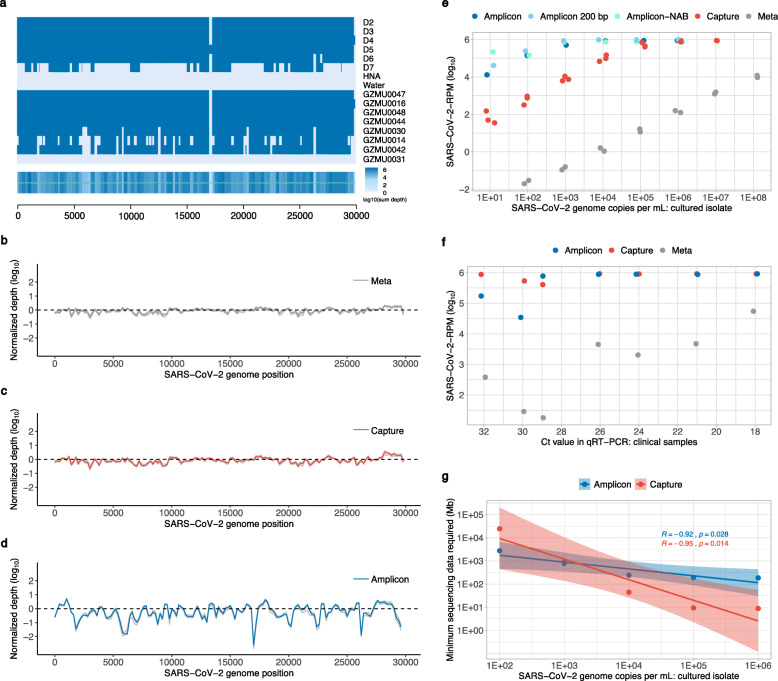
Sequencing coverage and depth of the cultured isolate and eight clinical samples. **a** Amplicon sequencing coverage by sample (row) across the SARS-CoV-2 genome. Dark blue, sequencing depth ≥ 100×; heatmap (bottom) sums coverage across all samples. HNA, negative control prepared from human nucleic acids; water, negative control prepared from nuclease-free water. Green horizontal lines on heatmap, amplicon locations. Overlap regions between amplicons range from 59 to 209 bp. **b**–**d** Normalized coverage across viral genomes of the clinical samples across methods. **e** SARS-CoV-2-RPM sequence plotted against genome copies per milliliter for the cultured isolate. Three independent experiments were performed for amplicon sequencing. Dark blue, ~ 400 bp amplicon-based sequencing including human and lambda phage nucleic acid background; soft blue, ~ 200 bp amplicon-based sequencing; fluorescent cyan, ~ 400 bp amplicon-based sequencing excluding human and lambda phage nucleic acid background (NAB); red, capture sequencing; grey, meta sequencing. **f** SARS-CoV-2-RPM (reads per million) sequence plotted against qRT-PCR Ct value for the clinical samples. Dark blue, amplicon; red, capture; grey, meta. **g** Estimated minimum amount of bases required by each method for high-confidence downstream analyses. Dark blue, amplicon; red, capture

Theoretically, amplicon sequencing should be the most sensitive and economical method among the three and is particularly suitable in an outbreak where viral isolates are highly related. Although, there are still potential pitfalls, for instance, the 40-cycle PCR in our workflow might augment trace amounts of SARS-CoV-2 cross-contamination. To ensure the confidence of the datasets, we included serial dilutions of the cultured isolate and negative controls prepared from nuclease-free water and human nucleic acids since the 1st PCR. All samples in ~ 400 bp amplicon-based sequencing exhibited > 99.5% coverage of 1× depth across the SARS-CoV-2 genome except for 1E+01 (95.23%), GZMU0031 (73.65%), HNA (6.17%), and water (60.24%) and > 97.00% coverage of 100× depth across the SARS-CoV-2 genome except for GZMU0030 (94.15%), GZMU0042 (88.17%), GZMU0014 (71.66%), D7 (39.49%), GZMU0031 (0.00%), HNA (0.00%), and water (0.00%), suggesting the primers were well designed and the positive datasets were reliable (Fig. 3a). We also set stringent and method-specific criteria to filter low-confidence sequencing reads and samples based on a set of controls (see the “Methods” section). Another pitfall is that amplification across the genome can hardly be unbiased, causing difficulties in complete genome assembly. Indeed, amplicon sequencing exhibited a lower level of uniformity compared with metatranscriptomic sequencing, in terms of coverage across the viral genomes from the cultural isolate and the clinical samples tested in our study (Fig. 3b, d; Additional file 2 Fig. S1). To our surprise, however, capture sequencing was almost as uniform as meta sequencing, demonstrating better performance than the previous capture method used to enrich ZIKV despite that SARS-CoV-2 genome is ~ 3-fold larger than ZIKV [44] (Fig. 3b, c). Two reasons among others were likely to be accountable to this improvement: (1) we utilized 506 pieces of 120 ssDNA probes covering 2× of the SARS-CoV-2 genome to capture the libraries, and (2) we employed the DNBSEQ sequencing technology that features PCR-free rolling circle replication (RCR) of DNA nanoballs (DNBs) [41, 42].

The sequencing results of amplicon and capture approaches revealed dramatic increases in the ratio of SARS-CoV-2 reads out of the total reads compared with meta sequencing, suggesting the enrichment was highly efficient—5596-fold in capture method and 5710-fold in amplicon method for each sample on average (Additional file 2 Table S2-S3). To further compare the sensitivity of different methods, we plotted the number of SARS-CoV-2 reads per million (SARS-CoV-2-RPM) of total sequencing reads against the viral concentration for each sample. Meta sequencing produced significantly lower SARS-CoV-2-RPM than the other two methods among clinical samples tested with a wide range of Ct values (Fig. 3e). The productivity was similar between the other two methods when the input RNA of the cultured isolate contained 1E+05 genome copies per milliliter and above (Fig. 3e). However, amplicon sequencing produced 10- to 100-fold more SARS-CoV-2 reads than capture sequencing when the input RNA concentration of the cultured isolate was 1E+04 genome copies per milliliter and lower, suggesting amplicon-based enrichment was more efficient than capture for more challenging samples (conc. ≤ 1E+04 genome copies per milliliter, or Ct ≥ 28.7) (Fig. 3e). Meta sequencing—as expected—produced dramatically lower SARS-CoV-2-RPM than the other two methods among clinical samples tested with a wide range of Ct values, whereas amplicon and capture were generally comparable to each other (Fig. 3f). Considering the costs for sequencing, storage, and analysis increase substantially with larger datasets, we tried to estimate how much sequencing data must be produced for each approach in order to achieve 10× depth across 95% of the SARS-CoV-2 genome, and the results can be found in Additional file 2 Table 3. As a practical, cost-effective guidance for future sequencing, we also assessed the minimum sequencing output required to pass the stringent filters (≥ 95% coverage and method-specific depth, see the “Methods” section) in our pipelines corresponding to different viral loads. We estimated that for high-confidence downstream analyses, amplicon sequencing requires at least 2757 to 186 megabases (Mb) for samples containing 1E+02 to 1E+06 copies of SARS-CoV-2 genome per milliliter, while capture sequencing requires 24,474 to 9 Mb for the same situation (Fig. 3g, Additional file 2 Table S4-S5).

### Investigation of inter- and intra-individual variations

**Fig. 4:**
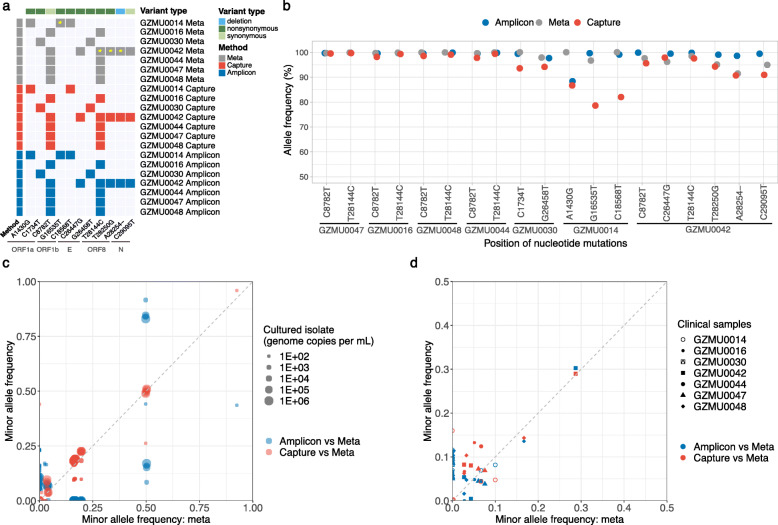
Between-sample and within-sample variants of SARS-CoV-2 detected across methods. **a** SNVs detected between clinical samples against a reference genome (GISAID accession: EPI_ISL_402119) [27]. Alleles with ≥ 80% frequencies were called. *SNVs verified by Sanger sequencing. **b** Allele frequencies of the identified SNVs. Dark blue, amplicon; red, capture; grey, meta. Minor allele frequencies detected in serial dilutions of the cultured isolate (**c**) and clinical samples (**d**) across methods. Dark blue, amplicon vs meta; red, capture vs meta. Minor alleles are defined with ≥ 5% and < 50% frequencies. Besides general quality filter, iSNVs had to pass depth and strand bias filter as described in the “Methods” section

To determine the accuracy of different approaches in discovering inter-individual genetic diversity, we tested each method in calling the single nucleotide variations (SNVs) and verified some of the SNVs with Sanger sequencing (Additional file 2 Fig. S2). Two to five SNVs were identified within each clinical sample, and in all the seven samples, SNVs identified by the three methods were concordant except that capture missed one SNV at position 16535 in GZMU0014 (Fig. 4a). We then investigated the allele frequencies of these sites across methods and found that alleles identified by capture sequencing displayed lower frequencies than the other two methods, especially for GZMU0014, GZMU0030, and GZMU0042 where the viral load was lower (Ct ≥ 29), which explained why capture sequencing neglected an SNV in our pipeline when the cutoff of SNV calling was set as 80% allele frequency (Fig. 4b). These data indicate that amplicon sequencing is more accurate than capture sequencing in identifying SNVs, especially for challenging samples.

To further determine the accuracy of different approaches in identifying SARS-CoV-2 iSNVs, we examined minor allele frequencies in serial dilutions of the cultured SARS-CoV-2 isolate and clinical samples. For serial dilutions of the cultured isolate, the minor allele frequencies detected in capture sequencing datasets were generally approximate to meta sequencing, while most allele frequencies in amplicon sequencing datasets deviated with those in meta sequencing (Fig. 4c). A similar pattern was shown for clinical samples, indicating that amplicon sequencing was unreliable of quantifying minor allele frequencies (Fig. 4d). Plotting allele frequencies against SARS-CoV-2 concentrations supported the above finding and further revealed that amplicon sequencing was unreliable of allele frequencies at all concentrations while capture sequencing was reliable at > 1E+03 genome copies per milliliter (Additional file 2 Fig. S3). Referring to the iSNV identified in clinical samples by meta sequencing, we then calculated the false positive rate (FPR) of minor alleles called by amplicon and capture methods. The FPR of minor alleles identified in amplicon sequencing was 0.74%, while that in capture sequencing was 0.02%. Together, these results suggest amplicon sequencing was not as accurate as capture sequencing in identifying minor alleles.

### Microbiome in clinical samples

In addition to target viral genome, metatranscriptomic sequencing has also allowed us to investigate RNA expression patterns of the overall microbiome and host content and thus suitable for discovering new viruses, distinguishing co-infections, and dissecting virus-host interactions. To explore the microbiota, we performed further metatranscriptomic analysis of the clinical samples. We were able to identify host nucleic acids in all of the samples, and over 95% of total reads were from the host in sputum, nasal, and throat samples (Additional file 2 Fig. S4a). Virus contributed to less than 5% of reads in anal swab and throat swab while more than 50% of reads in nasal swab (Additional file 2 Fig. S4b). These results suggest nasal swab could be the most ideal sample type for viral detection among the four sample types, which agrees with recent clinical evidence [45]. Previous studies have compared different sample types of other coronaviruses using qRT-PCR and found that nasopharyngeal aspirates and throat and nose swabs appear to be the most useful clinical specimens in the first 5 days of illness caused by SARS-CoV infection [46] and nasal swabs are the candidate sample of choice for detecting MERS-CoV using qRT-PCR technology in apparently healthy camels [47].

Among the viral reads, over 90% were Coronaviridae, which is consistent with clinical diagnostics (Additional file 2 Fig. S4c). Reads from other viruses were also identified, indicating further measurements could be taken to confirm if co-infection exists (Additional file 2 Fig. S4). Bacterial composition was also shown, providing support for scientific research, as well as for further confirmation of bacterial infection and antibiotics prescription (Additional file 2 Fig. S4d-f).

### Guidance for virus sequencing

Taken together, each sequencing scheme elaborated here for massively parallel sequencing of SARS-CoV-2 genomes has its own merits (Table 2). We hereby propose a reasonable, cost-effective strategy for sequencing and analyzing SARS-CoV-2 under different situations: (1) if one wants to study other genetic materials than the target viruses, or the viruses become highly diversified via recombinational events, or the viral load within the RNA sample is high (e.g., conc. ≥ 1E+05 viral genome copies per milliliter, or Ct ≤ 24.5), meta sequencing can be prioritized; (2) if one focuses on intra-individual variations for more challenging samples (e.g., conc. < 1E+05 and > 1E+03 viral genome copies per milliliter, or Ct > 24.5 and < 31.8), capture sequencing seems to be a justified choice; and (3) if identifying SNVs is the main purpose, the most convenient, economical strategy would be amplicon sequencing that can support analyses of samples containing lower than 1E+05 viral genome copies per milliliter, or Ct > 24.5.

**Table 2:** General characteristics of the three approaches employed in this study

	Metatranscriptomic sequencing	Hybrid capture-based sequencing	Multiplex PCR amplicon-based sequencing
**Sequencing objective**	Microbiome + human	Target genome	Target genome
**2nd strand synthesis**	Y	Y	N
**Fragmentation**	Y	Y	N
**Library preparation**	Y	Y	N
**PCR**	18 cycles	18 + 18 cycles	15 + 25 cycles
**Estimated time for library construction**	10.5 h	20.5 h	7.5 h
**Oligo synthesis**	*–*	120 nt × 506	40–60 nt × 2 × (113 + 14 + 10)
**Estimated cost per sample (USD)**^**a**^	112.86	65.14	48.00
**Estimated minimum data for downstream analyses (base level)**	> 10 Gb	Mb	Mb
**Uniformity**	High	Moderate	Low
**Sensitivity**	+	++	+++
**Accuracy (SNV)**	+++	++	+++
**Accuracy (iSNV)**	+++	++	+

^a^The price varies greatly with different sequencing output and in different regions

## Discussion

Sequencing low-titer viruses directly from clinical samples is challenging, especially for coronaviruses that are the largest among RNA viruses (~ 3-fold larger compared with ZIKV). Isolating viruses and enriching them in cell culture require high-standard laboratory settings and expertise apart from being time-consuming. The enrichment methods presented here have several advantages—to different degrees—over the other existing protocols [48, 49]. Firstly, the multiplex PCR protocol for ZIKV sequencing [48] and the Artic Network protocol for SARS-CoV-2 sequencing [49] require library preparation after PCR. Our amplicon method is more convenient since the barcoding and adaptor ligation steps are integrated into the PCR process; in other words, the PCR products are the library. Secondly, we adopt a set of controls to help us quantify viral load and identify potential contamination. During library construction for amplicon sequencing, each sample was mixed with standard lambda genomic DNA (external control), and the external control and the SARS-CoV-2 genomes are amplified at the same time. After sequencing, the viral load of SARS-CoV-2 is quantified based on the data from both external control and the target virus. We define a *C* = target viral load/(target viral load + external control load), and a *C* > 0.1% of negative groups (prepared from human nucleic acids and nuclease-free water) indicates severe contamination. Further, we consider a sample acceptable only when the *C* value of the sample is an order of magnitude higher than that of negative groups, for instance, if *C* < 0.01% for all negative controls and *C* > 0.1% for an experimental group. Finally, our work is the first that focuses around the use of BGI and MGI materials and platforms while previous protocols were mainly designed for Illumina or Oxford Nanopore Technologies (ONT).

Compared with direct metatranscriptomic sequencing, hybrid capture and amplicon sequencing methods are more sensitive but less accurate and neither of the two can be used to sequence highly diverse or recombinant viruses because the primers and probes are specific to known viral genomes. Although amplicon sequencing compromises its accuracy, it becomes the most convenient and economical method of all. Either or a combination of the approaches described here can be chosen to cope with various needs of researchers, e.g., metatranscriptomic sequencing data with insufficient coverage and depth can be pooled with hybrid capture data to generate high-quality assemblies [44]. From the perspective of virologists who conduct genomic studies of SARS-CoV-2, one dilemma is that there is hardly any standard of which sequencing method should be chosen for different samples or research purposes. Clinical specimens are precious, and it is unlikely to test each method on them. Of course, time and cost are also important factors that are needed to be considered. Therefore, in this work, we systematically examined the advantages and disadvantages of each method using different samples and proposed a guidance for rationally choosing the most suitable approach.

Moreover, we estimated the minimum sequencing output required for different samples using each method. This is another frequently encountered question, because larger output requires higher sequencing expenses, larger storage space, and more computing resources. The most cost-effective way is to generate the minimum amount of data needed for downstream analyses. Our work provides practical help for researchers to estimate how much data is necessary, although it varies with experimental procedures (e.g., total RNA extraction, rRNA depletion) and sample types (e.g., nasal swab typically requires less data than other sample types), and thus should be determined case by case.

Some advantages and disadvantages described above might be specific to the experimental workflows and bioinformatic pipelines presented in the current work, for instance, (1) the uniformity of amplicon sequencing can be improved by reducing the amount of cycles in the 1st PCR to 13 while increasing that in the 2nd PCR to 17 or increasing the molar ratios of primers targeting the region with low coverage, e.g., genomic position 16965–17246; (2) the amplicon sequencing is particularly convenient compared with previous counterparts since the standard fragmentation and library construction steps are omitted here by integrating adaptor and barcode ligation in the 2nd PCR and sequencing the amplicons using single-end 400 nt reads; (3) using less than 506 pieces of 120 ssDNA probes in hybrid capture may attenuate the sequencing coverage while decrease the uniformity; (4) metatranscriptomic sequencing was conducted with an ultra-high-throughput sequencing platform so that the successful rate was substantially higher than usual; and (5) the minimal amount of data necessary for analyzing the SARS-CoV-2 genome from clinical samples across methods can be deviated from that predicted by data from the cultured isolate, and this was possibly due to the fact that nucleic acid background from the host and other microbes varies (Additional file 2 Table S4-S5, Additional file 2 Fig. S4). Also, we do not consider the time spent in sequencing since the workflows can be easily adapted to various platforms including Illumina and ONT, besides DNBSEQ of MGI.

## Conclusions

All three methods can effectively obtain SARS-CoV-2 genome information from clinical samples and can be used to study genome variations of the virus. However, the sensitivity, accuracy, and cost of the three methods vary greatly, and thus, each method must be rationally chosen to cope with different research purposes and different clinical samples. This work offers practical guidance for genome sequencing and analyses of SARS-CoV-2 and other emerging viruses.

## Supplementary information

**Additional file 1: Table S1.** Genome positions of amplicons.

**Additional file 2: Fig. S1.** Normalized coverage across SARS-CoV-2 genomes of serial dilutions of the cultured isolate sequenced by multiple approaches. **Fig. S2.** Sanger sequencing results of SNVs in clinical samples. **Fig. S3.** Box-plot of allele frequencies against SARS-CoV-2 genome copies per mL. **Fig. S4.** Taxonomy of clinical samples by unbiased metatranscriptomic sequencing. **Table S2.** Capture sequencing data summary of serial dilutions of the cultured isolate and eight SARS-CoV-2 positive clinical samples collected from Guangzhou (PE100). **Table S3.** Amplicon sequencing data summary of serial dilutions of the cultured isolate and eight SARS-CoV-2 positive clinical samples collected from Guangzhou (SE400). **Table S4.** Estimated minimum amount of sequencing data required to achieve ≥10X sequencing depth and ≥ 95% coverage for different methods. **Table S5.** Estimated minimum amount of sequencing data required to achieve high-confidence variants calling analyses for different methods. **Table S6.** Bioinformatic tools and parameters used in this study.

**Additional file 3: Table S7.** Genome sequences acknowledged.

## Data Availability

The data that support the findings of this study have been deposited into CNSA (CNGB Nucleotide Sequence Archive, https://db.cngb.org/cnsa/) of CNGBdb with project IDs CNP0000951 [50] and CNP0000955 [51]; into GISAID (https://www.gisaid.org/, registration required) with accession numbers EPI_ISL_414663, EPI_ISL_414686, EPI_ISL_414687, EPI_ISL_414688, EPI_ISL_414689, EPI_ISL_414690, EPI_ISL_414691, and EPI_ISL_414692 [52]; and into NCBI with accession numbers MT568634, MT568635, MT568636, MT568637, MT568638, MT568639, MT568640, MT568641 [53] and PRJNA637515 [54]. The software and parameters used in data analyses during the current study are summarized in Additional file 2 Table S6.

## Abbreviations

MPS: Massively parallel sequencing
COVID-19: Coronavirus disease 2019
SARS-CoV: Severe acute respiratory syndrome coronavirus
MERS-CoV: Middle East respiratory syndrome coronavirus
BALF: Bronchoalveolar-lavage fluid
HAE: Human airway epithelial
iSNV: Intra-individual single nucleotide variation
Ct: Cycle threshold
RCR: Rolling circle replication
DNB: DNA nanoball
RPM: Reads per million
UDI: Unique dual indexing
HNA: Human nucleic acid
NAB: Nucleic acid background
